# Managing weight and glycaemic targets in people with type 2 diabetes—How far have we come?

**DOI:** 10.1002/edm2.330

**Published:** 2022-03-17

**Authors:** Matthias Blüher, Antonio Ceriello, Melanie Davies, Helena Rodbard, Naveed Sattar, Oliver Schnell, Elena Tonchevska, Francesco Giorgino

**Affiliations:** ^1^ Medical Department III – Endocrinology, Nephrology, Rheumatology University of Leipzig Medical Center Leipzig Germany; ^2^ Helmholtz Institute for Metabolic, Obesity and Vascular Research (HI‐MAG) of the Helmholtz Zentrum München at the University of Leipzig Leipzig Germany; ^3^ Department of Cardiovascular and Metabolic Diseases IRCCS MultiMedica Milan Italy; ^4^ Diabetes Research Centre University of Leicester Leicester UK; ^5^ 573772 NIHR Leicester Biomedical Research Centre Leicester UK; ^6^ Endocrine and Metabolic Consultants Rockville Maryland USA; ^7^ Institute of Cardiovascular and Medical Sciences, British Heart Foundation Glasgow Cardiovascular Research Centre University of Glasgow Glasgow UK; ^8^ Sciarc GmbH Baierbrunn Germany; ^9^ Forschergruppe Diabetes e. V. Munich Germany; ^10^ Department of Emergency and Organ Transplantation, Section of Internal Medicine, Endocrinology, Andrology and Metabolic Diseases University of Bari Aldo Moro Bari Italy

**Keywords:** type 2 diabetes, weight loss, HbA1c, treatment

## Abstract

**Introduction:**

As the vast majority of people with type 2 diabetes (T2D) are also overweight or obese, healthcare professionals (HCP) are faced with the task of addressing both weight management and glucose control. In this narrative review, we aim to identify the challenges of reaching and maintaining body weight targets in people with T2D and highlight current and future treatment interventions.

**Methods:**

A search of the PubMed database was conducted using the search terms “diabetes” and “weight loss.”

**Results:**

According to emerging evidence, treating obesity may be antecedent to the development and progression of T2D. While clinical benefits typically set in upon achieving a weight loss of 3–5%, these benefits are progressive leading to further health improvements, and weight loss of >15% can have a disease‐modifying effect in people with T2D, an outcome that up to recently could not be achieved with any blood glucose‐lowering pharmacotherapy. However, advanced treatment options with weight‐loss effects currently in development including the dual GIP/GLP‐1 receptor agonists may enable simultaneous achievement of individual glycemic and weight goals.

**Conclusion:**

Despite considerable therapeutic progress, there is still a large unmet medical need in patients with T2D who miss their individualized glycemic and weight‐loss targets. Nonetheless, it is to be expected that development of future therapies and their use will favourably change the scenario of weight and glucose control in T2D.

## Graphical abstract

In this narrative review, we aim to identify the challenges of reaching and maintaining body weight targets in people with type 2 diabetes and highlight current and future treatment interventions including high‐dose glucagon‐like peptide‐1 receptor agonists (GLP‐1 RA) and dual glucose‐dependent insulinotropic polypeptide (GIP)/GLP‐1 RA that may enable simultaneous achievement of individual glycemic and weight goals.

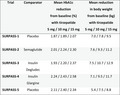

## 1 INTRODUCTION

Diabetes is a global public health burden, with type 2 diabetes (T2D) accounting for roughly 90% of all cases. This corresponds to approximately 537 million adults globally in 2021, and the number is projected to increase to 643 million by 2030 and to 784 million by 2045.^1^ In addition, according to the Centers for Disease Control and Prevention (CDC), almost 90% of individuals with T2D in the United States are also overweight (body mass index [BMI] ≥25 kg/m^2^) or obese (BMI ≥30 kg/m^2^).^2^ This shows that overweight and obesity are almost invariably associated with T2D.

Glycaemic control represents the primary target for people with T2D, regardless of the individual's body weight. Recent guideline recommendations suggest a glycated haemoglobin A1c (HbA1c) of less than 7% (53 mmol/mol) as a glycaemic target for the majority of adults without significant hypoglycaemia.^3^, ^4^, ^5^ However, glycaemic treatment goals should be individualized based on patient preferences and goals, risk of adverse effects of therapy (e.g., hypoglycaemia and weight gain) and patient characteristics, including frailty and comorbidities.^4^, ^5^ According to the American Diabetes Association (ADA), more stringent HbA1c targets may be recommended if they can be achieved safely and with acceptable burden of therapy, and less stringent targets (e.g., up to 8% [64 mmol/mol]) may be adequate for patients with limited life expectancy or in cases where the harms of treatment outweigh the potential benefits.^3^ Similarly, the shared European Society of Cardiology (ESC) and European Association for the Study of Diabetes (EASD) guideline^6^ supports individualized HbA1c targets, with HbA1c targets of 6.0%–6.5% (42–48 mmol/mol) in younger patients with a short diabetes duration and no evidence of cardiovascular disease, if achieved without significant hypoglycaemia. Less‐stringent HbA1c goals of up to 9% (75 mmol/mol) may be appropriate for elderly patients with long‐standing diabetes, limited life expectancy and frailty with multiple comorbidities.^6^

Nonetheless, achieving the individual glycaemic target is and remains even more challenging for overweight and obese patients. There is a positive and statistically significant association between excess body weight and inadequate glycaemic control.^7^ Consequently, overweight and obese people with T2D are less likely to meet their glycaemic targets compared to people with normal body weight.^7^ In this narrative review, we aim to determine the challenges of reaching and maintaining body weight targets in people with T2D and highlight treatment interventions that may enable simultaneous achievement of individual glycaemic and weight goals.

Overweight and obesity represent not just a frequent concomitant condition but are also amongst the leading causes of T2D, together with hereditary predisposition and lack of exercise.^8^, ^9^ Even among individuals with low genetic risk score and favourable lifestyle, obesity was associated with a >8‐fold increased risk of T2D compared with normal‐weight people.^10^ The rising prevalence of obesity worldwide, which increased threefold between 1975 and 2016,^11^, ^12^ is a global concern. In its latest report on the topic, the WHO announced that in 2016, more than 1.9 billion adults were overweight, and 650 million were obese, corresponding to 39% and 13% of the world population, respectively.^11^ Since overweight and T2D are closely interconnected, it is likely that the increasing global proportion of overweight and obese people will contribute to the increasing prevalence of diabetes in the years ahead. This is even more the case because of the recent COVID‐19 pandemic where many people have put on weight due to reduced exercise and overeating during lockdowns.^13^, ^14^, ^15^ More worryingly, small changes in body weight in relatively short periods can become permanent and lead to substantial weight gain over time.^16^

Targeting excess body weight may aid counteracting the epidemic of diabetes. Fat accumulation, predominantly in the abdominal or visceral region, can induce β‐cell dysfunction^17^ (also linked to excess fat in the pancreas), as well as excess liver fat and poorly regulated gluconeogenesis leading to the manifestation of hyperglycaemia in T2D.^18^, ^19^, ^20^, ^21^ Conversely, weight loss can reverse this process.^22^ Consistent evidence shows that obesity management can delay the progression from prediabetes to T2D^22^, ^23^, ^24^ and ameliorate hyperglycaemia in T2D.^22^ Moreover, in overweight or obese people with T2D, modest and sustained weight reduction reduced the need for glucose‐lowering medications.^22^ Even a modest intentional body weight reduction of 5% produces some clinically meaningful health benefits, which increase with more prominent weight loss.^25^, ^26^ In an analysis of 0.5 million people from a United Kingdom primary care database, individuals in the weight loss cohort had a median 13% weight loss resulting in T2D risk reductions of 41%, assuming a BMI of 40 kg/m^2^ before weight reduction.^27^ A weight loss of ≥15% can reverse T2D metabolic abnormalities and improve glucose control—an effect unattainable by any currently licensed glucose‐lowering treatment.^28^ Moreover, in the DiRECT clinical trial, intensive lifestyle changes with a low‐calorie diet and an average weight loss of about 10 kg led to T2D remission in around 46% of cases within one year^29^ and 36% after two years.^30^ Similar one year remission rates were seen in the DIADEM‐I trial conducted in Qatar.^31^

## 2 CHALLENGES IN ACHIEVING WEIGHT AND GLYCAEMIC TARGETS FOR INDIVIDUALS WITH T2D

### 2.1 Difficulty of reaching glycaemic targets

Getting T2D under control by reaching the glycaemic targets proves challenging for many patients. Typically, 40%–60% of people with T2D across several geographic regions and in both low‐ and higher‐income countries have suboptimal glycaemic control.^32^ Despite the accelerated rate of introduction of new medicine classes since the mid‐1990s,^33^ the percentage of people reaching the HbA1c targets of <7% has not substantially increased.^34^ Furthermore, reliance on pharmacotherapies only often fails to optimize glycaemic control. Despite the availability of insulin‐based therapies, roughly up to three quarters of patients failed to reach glycaemic targets.^35^ Long‐term maintenance of initial HbA1c level reduction proved an additional challenge when addressing glycaemic targets likely due to the progressive nature of beta‐cell dysfunction over time.^36^ Over a 10‐year period, a very modest improvement of 0.2 percentage points in HbA1c was demonstrated in T2D patients.^37^

### 2.2 Weight loss as a challenge in the context of T2D

Achieving glycaemic control represents just one more challenge for overweight and obese people with T2D that adds to the hurdle of reaching another target—weight loss. Data suggest that people with T2D experience more difficulty in trying to lose excess weight and maintain a healthy weight when compared to overweight people without diabetes regardless of the form of therapy utilized.^38^, ^39^ Moreover, one of the first long‐term studies comparing conventional therapies in the late 1990s and their effect on weight and glycaemia over a period of 15 years demonstrated weight regain within three years regardless of the applied treatment.^36^ Difficulty for overweight people with T2D in attaining both glycaemic targets and weight loss goals becomes even more urgent considering that the typical patient with T2D may have become one BMI unit (kg/m^2^) heavier over the course of 10 years.^37^ This excess weight gain may represent an even greater proportion of fat gain, as most people tend to lose muscle mass in older age, and this process is more accelerated in T2D.^40^, ^41^

Many obesity‐related risk factors depend mainly on body fat distribution rather than excess weight per se. The visceral fat, that surrounds organs within the abdominal cavity and rib cage, is associated with an increased risk of metabolic diseases.^42^ Although the underlying mechanism has not yet been fully understood, it is likely related to functional differences in subtypes of adipose tissue.^21^, ^43^ Visceral fat has been associated with higher metabolic activity and extent of inflammation.^44^ As a result, more free fatty acids^44^ and pro‐inflammatory adipokines^45^ are thought to be released to the bloodstream but further evidence proving such changes are causally linked to diabetes is needed.

### 2.3 Existing burdens in obesity management

Why does the challenge of achieving the respective targets persist? Potential explanations are likely to be multifactorial and linked to a number of patient‐, physician‐ and treatment‐related factors.

#### 2.3.1 Maintaining weight reduction, avoiding body weight variability

An existing burden is the frequent failure to maintain body weight reduction over time with obesity interventions, typically resulting in rapid weight loss followed by gradual regain.^46^, ^47^ A meta‐analysis of 29 long‐term weight loss studies found that just 23% of initial weight lost was maintained after 4 or 5 years.^48^ In the long term, obesity prevention and treatment strategies proved effective to a limited extent with, speculatively, hormonal, metabolic and neurochemical adaptations defending against weight loss and promoting weight regain.^49^

The failure to maintain weight loss may be partially explained by metabolic adaptation. Metabolic adaptation is a survival mechanism which acts to counteract weight loss and is thought to contribute to weight regain.^50^, ^51^ Since overweight and obese people typically burn more calories than normal‐weight individuals, their total energy expenditure significantly declines as people lose weight.^52^ The reduction in energy expenditure as well as the increased levels of hunger hormones and reduction in satiety hormones represent examples of metabolic adaptations in response to weight loss.^53^ Nonetheless, the effect of metabolic adaptation remains a controversial topic with recent studies showing that metabolic adaptation does not predict weight regain at up to two years of follow‐up.^54^, ^55^

The body weight variability (BWV) after weight loss should also be addressed since it is associated with greater cardiovascular risk in people with T2D, as shown in post hoc analyses of clinical trials.^56^, ^57^ In a real‐world study with Asian patients with T2D, BWV was associated with higher risks of myocardial infarction, stroke and all‐cause mortality.^58^ A recent study^59^ using data from the Swedish National Diabetes Register of 100,576 people with T2D and without prevalent cardiovascular diseases at baseline evaluated the link between visit‐to‐visit BWV and the risk of cardiovascular complications in a Caucasian population. High BWV predicted the development of cardiovascular complications such as non‐fatal myocardial infarction, non‐fatal stroke and all‐cause mortality in T2D.^59^ These studies further suggest that any weight loss strategy in people with T2D should be aimed at maintaining the reduction in the long term and avoiding body weight oscillations.

#### 2.3.2 Lack of education and clinical inertia

A lack of diabetes‐related education may contribute to the failure to achieve patient's treatment goals.^32^ For instance, 20% of the healthcare professionals (HCP) surveyed in the DAWN2 study reported that they had received no formal postgraduate education regarding diabetes.^60^ A survey indicated knowledge gaps in 46% of primary care physicians in Australia regarding the medical management of T2D.^61^ Underestimating the health consequences of obesity, which is yet not ubiquitously considered as a disease amongst HCPs but instead regarded simply as a failure to commit to a healthy lifestyle,^62^ may also represent an educational gap. A popular misconception is that a temporary change to better diet and more physical activity will reverse obesity, suggesting a common failure to recognize modern concepts in regulation of energy metabolism and body weight management.^62^ As a result, initially successful weight loss is frequently followed by a phase of weight regain.^63^, ^64^, ^65^, ^66^

Up to half of people with T2D appear to be inadequately treated due to clinical inertia and other reasons for underuse of intervention by their healthcare providers.^32^, ^67^, ^68^ Extended periods of ‘mild’ hyperglycaemia are often accepted by HCP.^32^ As a result, T2D patients often continue in poor glycaemic control without appropriate changes in therapy.^32^, ^69^, ^70^ Early exposure to inadequate glycaemic control in people with T2D can result in a significantly increased risk of myocardial infarction, heart failure, stroke or composite cardiovascular events.^71^ Substantial delays of a median of 1.6–2.9 years or 6.9–7.2 years in intensifying treatment by adding a second or third oral agent or insulin, respectively, were reported despite persistently high glycaemic levels.^72^ Early clinical inertia and delays in achieving a desired level of glycaemic control have been associated with increased probability of the patient failing to achieve their glycaemic targets later in the disease process.^69^, ^73^ The opposite has also been reported—treatment modification in patients with elevated HbA1c after 6 months reduced therapeutic inertia and was predictive of better long‐term glycaemic control.^74^

Clinical inertia is also a barrier to effective weight management. It presents a failure to start or intensify treatment and a missed opportunity to prevent complications at early stages (i.e., the progression from prediabetes to T2D) or reduce the risk of long‐term complications (i.e., cardiovascular events).^75^ Much of the inertia in addressing obesity can be attributed to the prevailing and persistent framing of obesity as matter of personal responsibility.^62^, ^76^

The causes of clinical inertia are multifactorial and occurring at the level of the practitioner, patient and/or healthcare system.^77^ Regardless of the cause, there is a pressing need to address clinical inertia. Multidisciplinary teams, a coordinated chronic care model, including self‐management and decision support, delivery system design, clinical information systems, community resources and policies, may counteract clinical inertia by promoting interaction between more empowered patients and better prepared HCP.^4^, ^5^

In addition to these multiple factors leading to a reduced likelihood of achieving individualized targets, one of the most important factors may be underestimating the interconnection of T2D and obesity. A current hypothesis is that treating obesity may be antecedent to the development and progression of T2D, such that weight loss may result in better glycaemic control also prospectively.^28^ If this hypothesis were correct, this would imply that obesity is often neglected in T2D when it should be the first priority for intervention.

## 3 TREATMENT OF OBESE PEOPLE WITH T2D

### 3.1 Enhancing weight loss with current therapeutic options

As discussed above, weight management emerges as another important target as glycaemic control for a majority of people with T2D. Various treatments aid achieving glycaemic targets and enhancing body weight loss to a different extent. Here, lifestyle interventions, weight loss medications, anti‐diabetes pharmacotherapies and bariatric surgery will be discussed as well as their effects on both targets.

#### 3.1.1 Diet, physical activity and behavioural intervention

Lifestyle modifications such as diet and increased physical activity have been established as a cornerstone of the treatment of T2D and obesity. Moreover, it has been recommended as a first‐line strategy by guidelines for management of both diabetes and obesity.^4^, ^5^, ^22^, ^78^, ^79^ The aim is management and reversal of excess weight that can lead to better glycaemic control. The approach should be a high‐quality hypocaloric diet, which promotes patient's adherence accompanied by a minimum of 150 min of moderate activity per week.^80^

The feasibility of these recommendations was demonstrated in the Look AHEAD clinical trial.^81^ In that study, intensive lifestyle intervention resulted in clinically meaningful weight loss (≥5%) in 50% of people with T2D, and approximately 26% maintained a body weight loss of ≥10% at year 8.^81^ Moreover, intensive dietary interventions with low‐^29^, ^30^ and very‐low‐calorie diets^82^, ^83^ have been shown to achieve substantial reduction of HbA1c and sustained T2D remission in obese people with T2D. However, according to the ADA, such structured, low‐calorie diets should be prescribed only for carefully selected patients by well‐trained and experienced practitioners with close monitoring.^22^ For the vast majority of obese people with T2D, significant weight loss is feasible with lifestyle programs that achieve a 500–750 kcal/day energy deficit,^22^ somewhat regardless of macronutrient composition.^84^, ^85^ Accordingly, dietary choice should be individually tailored to the patient's preferences and nutritional needs.^22^, ^86^

Besides energy intake in the form of calories, energy expenditure is the other important determinant in the body's energy balance. Thus, weight loss can be attained by selective increase of energy expenditure utilizing physical activity,^87^ although most people would need to exercise for several hours per week to achieve such weight loss which is unfeasible for most. Moreover, regular exercises could present a physical burden on people with T2D due to their often low physical performance threshold.^88^ Exercise or increasing activity is, however, a very effective intervention to help prevent or minimize weight (re)gain in adults.^89^ Weekly moderate to vigorous physical activity is recommended for T2D management.^4^, ^5^, ^90^ Encouraging high levels of physical activity (200–300 min/week) after achieving short‐term weight loss goals is also recommended,^22^ although hard to achieve for many. For the large proportion of people with T2D who are ageing, currently sedentary, overweight or obese, deconditioned or unable to embark upon structured exercise, ‘sitting less’ may prove an alternative behavioural strategy. In a recent experimental study in postmenopausal women, a significant improvement in peripheral insulin sensitivity in the sitting less (~13%) and the exercise regimen (~20%) has been reported, compared with the sitting regimen.^91^ Encouragingly, these results confirmed earlier findings in which breaking sitting with standing and light‐intensity walking effectively improved 24 h glucose levels and insulin sensitivity in elderly people with T2D.^92^ Therefore, people should be encouraged to find some physical activity that they enjoy and are likely to sustain or to vary physical activities to have more tools to help maintain higher habitual activity levels. Yet, physical activity represents just one component of long‐term (≥1 year) weight‐maintenance programs, which additionally provide regular contact and support.^22^ Moreover, combining dietary interventions and physical exercise improves hyperglycaemia and reduces cardiovascular risk factors more than diet or physical activity alone.^84^

#### 3.1.2 Weight loss medications

Obesity pharmacotherapy is a valuable option in patients with a BMI >30 kg/m^2^ or with a BMI >27 kg/m^2^ in the presence of weight‐related comorbidities, such as diabetes, hypertension and dyslipidemia. It has been recommended as an adjunct to lifestyle modifications by the American Association of Clinical Endocrinologists and the American College of Endocrinology^78^ with the overall rationale to aid adherence to dietary recommendations, in most cases by regulating appetite or satiety.^22^, ^93^

A few anti‐obesity agents for long‐term use have been approved by the U.S. Food and Drug Administration (FDA) and/or the European Medicines Agency (EMA) (Table 1). Among them are orlistat, phentermine/topiramate extended release (ER), naltrexone (ER)/bupropion (ER) and the glucagon‐like peptide‐1 receptor agonists (GLP‐1 RAs) liraglutide 3 mg and semaglutide 2.4 mg.^94^ All of them result in clinically meaningful weight loss and improve glycaemic control with data in patients completing at least one year of drug treatment.^93^ The most recently approved treatment, semaglutide 2.4 mg, has been associated with the greatest weight loss compared with the other approved agents.^95^ Semaglutide has been tested within the STEP clinical trials to promote weight loss in overweight or obese subjects without^95^ and with T2D.^96^ Notably, both GLP‐1 RAs are diabetes medications explicitly approved for weight management in non‐diabetic patients by appetite suppression.^95^, ^97^

**TABLE 1:** FDA and/or EMA‐approved medications for chronic weight management

Medication name	Pharmacologic class	Typical adult maintenance dose	Mean reduction in body weight from baseline (%)^a^
Orlistat	Lipase inhibitor	60 mg (OTC), 120 mg (Rx), three times daily, PO	2.9^169^
Phentermine/topiramate ER	Sympathomimetic amine anorectic/antiepileptic	7.5 mg/46 mg (max dose 15 mg/92 mg), daily, PO	9.8 (15 mg/92 mg) 7.8 (7.5 mg/46 mg)^170^
Naltrexone ER/bupropion ER	Opioid antagonist/antidepressant	16 mg/180 mg, twice daily, PO	5.0^171^
Liraglutide	GLP‐1 RAs	3 mg daily, SQ	8.0^97^
Semaglutide	GLP‐1 RAs	2.4 mg, weekly, SQ	9.6 – with T2D^96^ 14.9 – without T2D^95^

Abbreviations: ER, extended release; OTC, over the counter; PO, oral; Rx, prescription; SQ, subcutaneous.

^a^ Results from clinical trial combining lifestyle modifications.

Another treatment approach for weight management is the long‐acting amylin analogue cagrilintide. A phase 2 trial with cagrilintide 0.3–4.5 mg studied its effect on weight loss in people with overweight and obesity compared to liraglutide 3.0 mg, and to placebo. Mean percentage weight reductions after 26 weeks of treatment were greater with all doses of cagrilintide (6.0%–10.8%) versus placebo (3.0%), and also with the highest dose cagrilintide 4.5 mg (10.8%) versus liraglutide 3.0 mg (9.0%). Moreover, with the highest cagrilintide dose, 88.7%, 53.5% and 18.7% of people achieved weight loss of at least 5%, 10% and 15%, respectively. Gastrointestinal (GI) disorders and administration‐site reactions were the most frequent adverse events, occurring in 41%–63% of the cagrilintide groups, compared with 60% of the liraglutide group and 32% of people taking placebo.^98^

#### 3.1.3 Glucose‐lowering pharmacotherapies with weight loss effects

In addition to pharmacotherapies addressing obesity directly, there are anti‐diabetes medications that promote weight loss. Glucose‐lowering medications that are weight‐neutral or promote weight loss are recommended when treating people with T2D and overweight or obesity.^4^, ^5^, ^22^, ^79^ The therapies associated with varying degrees of weight reduction include metformin, α‐glucosidase inhibitors (AGI), amylin mimetics, sodium–glucose cotransporter 2 inhibitors (SGLT2i) and GLP‐1 RAs.^22^ In the following section, the drug classes associated with weight loss will be reviewed.

##### Metformin

Metformin remains the initial pharmacologic agent of choice for the treatment of patients with T2D unless there are contraindications.^4^, ^5^, ^79^, ^99^ Predominantly prescribed as a monotherapy in combination with lifestyle modifications, metformin may be combined with other agents in the presence of cardiovascular or minor renal complications, or when it is necessary to improve glycaemic control or promote weight loss.^99^ Metformin belongs to the biguanides family and has the ability to decrease hepatic glucose production and intestinal absorption of glucose, as well as to exert insulin‐like effects by increasing peripheral glucose uptake and utilization.^100^ Treatment with metformin led to decreased HbA1c by approximately 1 percentage point compared to placebo after 3 months of therapy.^101^ Moreover, approximately half of the studies conducted to date have shown significant but modest reductions in body weight with metformin compared with baseline or comparators, and weight changes of +1.5 to −2.9 kg in insulin‐naïve patients have been reported.^102^

##### α‐glucosidase inhibitors

AGIs, or α‐glucosidase inhibitors, are another class of oral glucose‐lowering agents with a relatively limited use in the clinic. They inhibit intestinal α‐glucosidase activity and delay the absorption of carbohydrates in the gastrointestinal tract, which in turn slows the spike in postprandial glucose.^43^ They demonstrate a HbA1c‐lowering effect with associated reduction of 0.64 percentage points compared with placebo,^103^ as well as a modest weight loss between −0.43 and −1.80 kg^43^ in patients with T2D.

##### Amylin mimetics

Pramlintide, an amylin mimetic, has been approved only in the United States for people with T1D and T2D, predominantly in combination with insulin therapy. This agent is a peptide with a dual function of a neuropeptide and a circulating endocrine hormone secreted from islet β cells. After subcutaneous injection, the peptide activates amylin receptors and this results in suppressed glucagon secretion, slowing of gastric emptying and increased satiety.^104^ In people with T2D, up to 150 µg pramlintide three times daily had a mean HbA1c‐ and body weight‐lowering effect compared to placebo of up to 0.4 percentage points and up to 2.5 kg, respectively.^105^, ^106^ A systematic review of studies until 2009 demonstrated that people with T2D experienced modest weight loss of up to 3.7 kg after 16 weeks of pramlintide 120–240 µg administered three times daily.^107^ The most commonly reported adverse events were nausea of any severity and hypoglycaemia in people randomized to pramlintide vs. control.^105^, ^106^ Reports of nausea occurred predominantly in the early weeks of therapy, were mild‐to‐moderate intensity, were dose‐dependent, and subsided over time.^108^ With regard to hypoglycaemia, adding pramlintide to an insulin therapy carried the risk of severe hypoglycaemia in people with T2D vs. placebo (0.9 vs. 0.3 events/patient‐year in the first 4 weeks of combination treatment) if concomitant insulin use was not proactively reduced.^109^ These findings indicated that side effects can be managed by gradual titration program at pramlintide initiation.^108^ However, due to its side effects, frequent dosing schedule of daily injections as well as the comparatively modest effect on glycaemic control and body weight, pramlintide clinical uptake has been limited.

##### SGLT2i

Other glucose‐lowering agents introduced subcutaneously are the sodium–glucose cotransporter 2 inhibitors (SGLT2i), that is, canagliflozin, dapagliflozin, empagliflozin and ertugliflozin. This class prevents glucose reabsorption in the kidneys, resulting in an increased glucose excretion.^110^ In turn, renal glucose excretion is thought to facilitate weight loss, both through caloric deficit and fluid loss due to increased osmotic diuresis.^111^ In a meta‐analysis, SGLT2i have been shown to lower HbA1c by approximately 0.7 percentage points compared with placebo, with canagliflozin resulting in 0.85 percentage points reduction.^112^ Meta‐analyses have revealed a greater body weight loss in patients with T2D treated with SGLT2i compared with placebo^113^ or other anti‐diabetes medications (sulfonylureas, thiazolidinediones and insulin glargine),^114^ ranging −2.0 to −2.3 kg or −3.81 to –4.61 kg, respectively. In addition to promoting weight loss, SGLT2i exerted beneficial effects on blood pressure, as well as reduction of cardiovascular (in particular heart failure) and renal events.^113^, ^115^ Thus, a therapy with SGLT2i or GLP‐1 RAs is recommended as a second‐line therapy after metformin when there is a compelling need to minimize weight gain or to promote weight loss.^4^, ^5^, ^79^ Moreover, the use of SGLT2i is recommended for patients with T2D who have established atherosclerotic cardiovascular disease or indicators of high risk, established renal disease, heart failure^4^, ^5^, ^79^, ^99^ or if there is a compelling need to minimize hypoglycaemia.^4^, ^5^, ^79^ Nevertheless, compared with placebo, treatment with SGLT2i increased the risk of urinary and genital tract infections by a factor of 1.14 and 4.34, respectively.^113^

##### GLP‐1 RAs

As discussed above, agents of the glucagon‐like peptide‐1 receptor agonists (GLP‐1 RAs) class have been approved for weight management in people with or without T2D. Historically, the class has been tested as a glucose‐lowering therapy. Among its approved agents are liraglutide, dulaglutide and the most potent one—semaglutide. They mimic the GLP‐1 incretin hormone which is released in the gastrointestinal tract in response to nutrient intake. The GLP‐1 RAs bind to the GLP‐1 receptors on beta cells resulting in enhanced glucose‐dependent insulin secretion, suppressed glucagon secretion, increased resistance to apoptosis and possibly induction of proliferation.^116^ In addition, GLP‐1 RAs act in the central nervous system to decrease appetite, promoting satiety and suppressing energy intake.^117^ Further, GLP‐1 RAs target the gastrointestinal tract and slow gastric emptying, which in turn delays intestinal glucose absorption.^118^ Taking these multiple effects into account, GLP‐1 RAs reduce both fasting and postprandial blood glucose levels in a glucose‐dependent manner.^118^ The most frequently reported adverse events associated with GLP‐1 RAs are nausea, vomiting and diarrhoea.^43^ Their cause is thought to be the effects of these agents on the central nervous system as well as a result of delaying gastric emptying in some individuals.^118^

Studies demonstrated the efficacy of GLP‐1 RAs in reducing HbA1c by up to 1.9 percentage points compared with baseline and promote weight loss of up to 6.9 kg.^43^, ^119^, ^120^, ^121^ There were also significant reductions in cardiovascular events and some renal outcomes in the absence of hypoglycaemia due to their glucose‐dependent mechanism of action.^43^, ^122^ While all approved GLP‐1 RAs have high glucose‐lowering and weight loss efficacy, there is variation within the drug class.^123^ Structural differences among GLP‐1 RAs influence duration of action, and their formulation and dosing may affect efficacy in reducing blood glucose and body weight, as well as side effect profile and cardiovascular effects.^124^ However, an increased dosage of multiple approved GLP‐1 RAs has been studied in recent trials. The AWARD‐11 trial^125^ compared dulaglutide at doses of 3.0 and 4.5 mg versus the approved dose of 1.5 mg in people with T2D inadequately controlled with metformin. Indeed, escalation to a higher dose of dulaglutide provided clinically relevant, dose‐related reductions in HbA1c (1.72 percentage points vs. 1.61 percentage points vs. 1.55 percentage points with dulaglutide 4.5, 3.0 and 1.5 mg, respectively) and body weight (4.9 kg vs. 4.0 kg vs. 3.4 kg with dulaglutide 4.5, 3.0 and 1.5 mg, respectively) at 52 weeks with a similar safety profile.^125^ Another example is the weekly injectable semaglutide, currently approved at doses of up to 1.0 mg for people with T2D and up to 2.4 mg for obesity management. In the STEP trials, semaglutide plus a lifestyle intervention was tested at the higher dose of 2.4 mg/week, specifically for promoting weight loss, regardless of the presence of T2D (Table 2).^126^ Adverse effects were in line with those expected for a GLP‐1 receptor agonist, with mild‐to‐moderate GI events being the most common.^95^, ^96^, ^127^, ^128^

**TABLE 2:** STEP‐program phase 3 trials with efficacy results

Trial	Trial objective	*N*	EOT (weeks)	Comparator	Mean HbA1c at baseline (%)	Mean BMI at baseline (kg/m^2^)	Mean reduction in body weight from baseline (%)
STEP 1^95^	WM	1961	68	Placebo	5.7	37.9	14.9
STEP 2^96^	WM in T2D	1210	68	Semaglutide 1.0 mg or placebo	8.1	35.7	9.6
STEP 3^127^	WM with IBT	611	68	Placebo	5.7	38.0	16.0
STEP 4^128^	Sustained WM	902	68	Placebo for 48 weeks after 20 weeks of semaglutide 2.4 mg	5.7	38.3	17.4

Abbreviations: EOT, end of treatment; HbA1c, haemoglobin A1c; IBT, intensive behavioural therapy; T2D, type 2 diabetes; WM, weight management.

Both GLP‐1 RAs with good efficacy for weight loss and SGLT2i are recommended as a second‐line monotherapy when there is a compelling need to minimize weight gain, or to promote weight loss.^4^, ^5^, ^79^, ^99^ Moreover, the use of GLP‐1 RAs is recommended for people with T2D who have established atherosclerotic cardiovascular disease or indicators of high risk, established renal disease, or if there is a compelling need to minimize hypoglycaemia.^4^, ^5^, ^79^, ^99^

#### 3.1.4 Bariatric surgery

A landmark study published in 1995 introduced bariatric surgery as a long‐term treatment for obesity and T2D. It showed for the first time that a gastric bypass operation could normalize glycaemia, insulin function and HbA1c levels for 14 years of follow‐up in 83% of people with diabetes with a BMI ≥35 kg/m^2^. Hence, the procedure resulted in significant, consistent and durable glucose control in addition to weight loss.^129^ Meanwhile, multiple bariatric surgery approaches are available including gastric banding, sleeve gastrectomy, gastric bypass, biliopancreatic diversion and others.

Currently, bariatric surgery is considered the gold standard treatment for severe obesity (BMI ≥40 kg/m^2^) due to its high efficacy in terms of weight loss, duration of effectiveness and improvement of T2D.^22^ It is also recommended by the ADA for patients with a BMI 35.0–39.9 kg/m^2^ with inadequately controlled hyperglycaemia despite optimal medical therapy.^22^ Almost 70% of patients experience complete T2D remission within 5 years following surgery, with a median duration of remission of 8.3 years.^130^

In recent studies, significantly more obese patients with uncontrolled T2D achieved glycaemic control after a year of medical therapy plus bariatric surgery.^131^ Another trial^132^ confirmed that severely obese patients (BMI ≥35 kg/m²) with T2D achieved better glycaemic control after bariatric surgery than with medical therapy. At 2 years, diabetes remission (defined as a fasting plasma glucose level of less than 100 mg per decilitre (5.6 mmol per litre) and a HbA1c of less than 6.5% for at least 1 year without active pharmacologic therapy) was not observed in any patients in the medical therapy group compared to 75% in the gastric bypass group and 95% in the biliopancreatic diversion group. A significant difference in extent of weight loss between the surgical and medical therapy groups was also observed, with no significant difference between the two surgical groups.^132^ Further, the 10‐year follow‐up data indicated that 25% of patients in the gastric bypass group and 50% in the biliopancreatic diversion group remained in remission.^133^

Unfortunately, about one third of patients in remission relapsed (defined as restarting diabetes medication and/or one or more HbA1c measures ≥6.5%) within 5 years of initial remission. This suggests that surgery is associated with durable remission of T2D in many but not all people with diabetes living with severe obesity.^130^ For multiple reasons, including cost, limited access to care and concerns about adverse events, bariatric surgery has been limited to a small proportion of those eligible for the procedure.^87^

### 3.2 Potential future therapeutic options

In addition to the available therapies, multiple others are in development. Since it appears that a combination therapy was superior to monotherapy in newly diagnosed T2D,^134^ one of the preferred combination partner for next‐generation therapies could be the GLP‐1 RAs due to its established robust improvement in glycaemic control and weight loss and its cardioprotective effects.^135^ An alternative strategy is the creation of peptide combinations with complementary modes of action such as unimolecular co‐agonists and triagonists with GLP‐1 again emerging as an ideal partner.^136^

#### 3.2.1 GLP‐1 RAs in combination with SGLT2i

Two prospective studies looked into the combination of GLP‐1 RAs with an SGLT2 inhibitor. In the DURATION‐8 study, combined therapy with the GLP‐1 RAs exenatide and the SGLT2i dapagliflozin was observed to reduce HbA1c by 2 percentage points and simultaneously produced a weight loss of 3.4 kg. Both targets were superior to those obtained with monotherapy with either agent.^137^ On the other hand, the AWARD‐10 study demonstrated an up to 1.34 percentage points HbA1c reduction and a weight loss of 3.1 kg when the GLP‐1 RAs dulaglutide 1.5 mg was added to the treatment with SGLT2i (with or without metformin).^138^ Moreover, both dual treatment regimens improved cardiovascular risk factors and were well tolerated. The most recent trial, SUSTAIN 9, investigated the combined treatment with the GLP‐1 RAs semaglutide 1.0 mg as an add‐on therapy to SGLT2i in patients with inadequately controlled T2D. Patients randomized to the combination treatment had a significant reduction in HbA1c (1.42 percentage points) and bodyweight (3.81 kg) and showed good tolerability.^139^ A meta‐analysis^140^ of four randomized controlled trials compared a therapy with SGLT2i to combination treatment of GLP‐1 RAs as add‐on to SGLT2i. The GLP‐1RA/SGLT2i combination was associated with greater reduction in HbA1c (0.74 percentage points) and body weight (1.61 kg), and similar incidence of hypoglycaemia compared to SGLT2i alone.^140^ These results suggest that the efficacy of combined GLP‐1 RAs/SGLT2i therapy is partially additive in lowering HbA1c level and body weight.

#### 3.2.2 Dual GIP/GLP‐1 RAs

**FIGURE 1:**
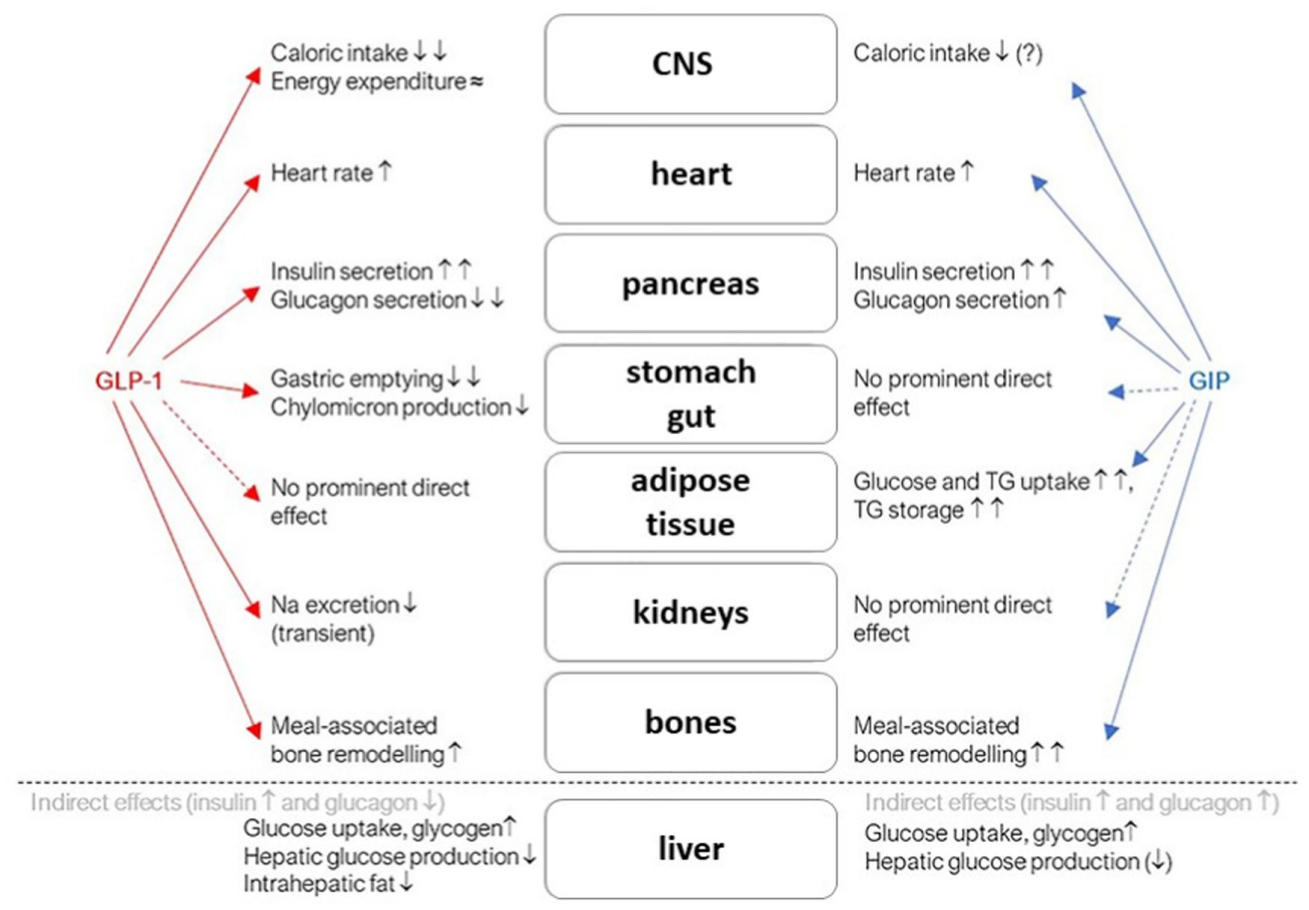
Overview on biological GIP and GLP‐1 effects at the organ/tissue level^147^

By definition, incretin hormones are characterized by low baseline concentrations in the fasting state and substantial increases after food intake. There are two known incretins. The glucose‐dependent insulinotropic polypeptide (GIP) is a peptide synthesized and secreted mainly by K cells in the duodenum and proximal jejunum, and GLP‐1 is a peptide synthesized and secreted mainly by L cells in the small and large intestine. In addition, both incretins as well as their receptors are also expressed in the central nervous system (CNS).^141^, ^142^ Together, they are responsible for the ‘incretin effect’, which refers to the observation of a twofold to threefold increase in insulin secretion following oral glucose ingestion compared with a corresponding intravenous glucose administration (Figure 1).^143^ Both GIP and GLP‐1 secretion are stimulated mainly by the ingestion and absorption of carbohydrates and triglycerides or their digestion products and, to a lesser extent, by proteins or amino acids. In people with T2D, plasma concentrations of GIP are higher after an oral glucose load or after a meal than in control subjects without diabetes.^144^

##### Insulin secretion and the incretin effect

In healthy humans, both GIP and GLP‐1 stimulate insulin secretion in a glucose‐dependent manner, such that plasma glucose concentrations determine the degree of the insulin secretagogue effect in individuals exposed to GIP and/or GLP‐1.^145^ The incretin effect contributes significantly to important mechanisms required for the maintenance of normal glucose tolerance. A reduction in the incretin effect is usually associated with impaired oral glucose tolerance, and it is reduced in people with T2D due to defects in GLP‐1/GIP levels and/or action.^146^

Proposed mechanisms for the loss of the incretin effect include, first, a reduced response of incretin hormones to nutrients and, second, a reduction in the insulinotropic effect on pancreatic beta cells. The severely impaired insulinotropic effect of GIP is the main reason for the described reduced incretin effect in patients with T2D. The insulinotropic effect of GLP‐1 in people with T2D is slightly different, as its ability to stimulate insulin secretion in hyperglycaemia is largely preserved in T2D. Pharmacological doses of GLP‐1 RAs have been found to elicit insulinotropic effects. The combination of GIP and GLP‐1 tends to have a lower insulinotropic effect than the sum of the individual effects of GIP and GLP‐1 administered separately.^147^

##### Glucagon secretion

Whereas the stimulation of insulin secretion by GIP and GLP‐1 is characterized by great similarities in terms of their dose‐response characteristics and glucose dependence, there are distinguishing differences concerning glucagon secretion. While GLP‐1 suppresses glucagon secretion, GIP can stimulate glucagon secretion. GIP can stimulate glucagon secretion in people with T2D during hypoglycaemia.^146^ On the other hand, GLP‐1 suppresses glucagon concentration during hyperglycaemia but not during euglycaemia or hypoglycaemia. The combination no longer lowers glucagon concentration, suggesting an interaction between GIP and the suppression of glucagon secretion observed with GLP‐1 alone.^147^

##### Body weight regulation, food intake and energy expenditure

The potential influence of GIP and/or GLP‐1 on body weight regulation is another important biological activity that may represent a therapeutic potential for incretin hormones. Receptors for GIP and GLP‐1 have been identified in brain regions involved in the regulation of appetite, satiety, food/energy intake and energy expenditure. GIP and/or GLP‐1 receptors in other brain regions may be involved in anti‐apoptotic effects, synaptic plasticity, memory, reward functions and emotional responses, which could have beneficial effects on several neurodegenerative diseases.^147^

##### Effects on adipose tissue function

Glucose‐dependent insulinotropic polypeptide receptor density appears to decrease in people with obesity and may increase again after weight loss. It is hypothesized that the ability of GIP to target white adipose tissue and increase its lipid buffering capacity may protect against dietary fat ‘spillover’. Thus, combining the anorectic effect of GIP/GLP‐1 RAs with the peripheral effect of GIP to promote lipid storage in white adipose tissue may be advantageous over the mechanisms of current treatments for T2D, by enhancing both insulin secretion and insulin sensitivity.^148^ In addition, GIP is thought to be responsible for the incorporation of non‐esterified fatty acids into adipose tissue and probably influences fat deposition in other tissues, such as the liver.^149^

##### Gastric emptying

Gastric emptying is slowed by physiologic and pharmacologic doses of GLP‐1, with higher doses leading to complete cessation of gastric emptying. GLP‐1 RAs also delay gastric emptying.^150^ Both physiologic and pharmacologic concentrations of GLP‐1 reduce the rate of entry of nutrients into the bloodstream by reducing gastric emptying, which is an important mechanism for the control of postprandial hyperglycaemia and also for the satiating effect of this gut hormone. In contrast, GIP does not affect gastric emptying.^147^

##### Tirzepatide

A novel dual GIP and GLP‐1 RAs were developed to determine whether the metabolic effects of GIP add to the established clinical benefits of selective GLP‐1 RAs in T2D. Tirzepatide is a dual GIP/GLP‐1 RAs formulated as a 39‐amino acid synthetic linear peptide based on the native GIP sequence. It is attached to a 20‐carbon fatty acid moiety that binds to albumin, which extends its half‐life to 5 days, allowing once‐weekly dosing administered subcutaneously. Tirzepatide has comparable GIP receptor binding affinity to native GIP and five times lower GLP‐1 receptor affinity than native GLP‐1.^151^

##### SURPASS clinical trial program

The SURPASS clinical trial program aimed to evaluate the efficacy and safety of tirzepatide as a treatment to improve glycaemic control in people with T2D. The phase 3 SURPASS clinical trials include seven global trials including one CVOT trial, two Japanese trials and one Asia‐Pacific trial.^152^ These trials included patients who did not receive antihyperglycaemic therapy (patients treated with diet and lifestyle only) as well as patients who received various oral antihyperglycaemic agents (metformin, sulfonylureas, SGLT2 inhibitors and/or insulin). Some trials are placebo‐controlled, others have active comparators such as GLP‐1 RAs (dulaglutide and semaglutide), long‐acting insulin analogues (glargine and degludec) or short‐acting insulin analogues (lispro). The SURPASS trials evaluate once‐weekly tirzepatide doses of 5, 10 and 15 mg. It takes 4 weeks to reach the 5 mg dose, 12 weeks for the 10 mg dose and 20 weeks for the 15 mg dose. The primary endpoint for each of the studies is the change from baseline in HbA1c. The SURPASS clinical program included also the SURPASS‐CVOT—a large‐scale, randomized, double‐blind and phase 3 cardiovascular outcomes trial of tirzepatide evaluating both non‐inferiority and superiority of tirzepatide versus dulaglutide (1.5 mg weekly). The SURPASS‐CVOT study has randomized over 12,500 participants from 30 countries with T2D (age 40 years, HbA1c between 7.5% and 10.5%, BMI >25 kg/m^2^) and established atherosclerotic cardiovascular disease. Notably, one of the secondary endpoints of the CVOT study was the percentage of participants with weight loss of >10%.

In the completed SURPASS trials, treatment with tirzepatide at all doses (5, 10, 15 mg) demonstrated greater reductions in HbA1c (and achievement of HbA1c <7.0%) compared with placebo,^153^ semaglutide 1 mg,^154^ insulin degludec^155^ and insulin glargine,^156^ without increasing the risk of hypoglycaemia. Similarly, treatment with tirzepatide has been associated with greater weight loss and high achievement of weight loss goals (Table 3). In the SURPASS‐2 trial, tirzepatide at all doses was associated with a significantly higher proportion of patients achieving weight loss goals of >5% (65%, 76%, 80% with tirzepatide 5 mg, 10 mg, 15 mg, respectively, vs. 54% with semaglutide 1 mg), >10% (34%, 47%, 57% with tirzepatide vs. 24% with semaglutide 1 mg) and >15% (15%, 24%, 36% with tirzepatide vs. 8% with semaglutide 1 mg). Moreover, compared with semaglutide 1 mg treatment with tirzepatide was associated with greater reductions in HbA1c, body weight and blood pressure, as well as greater improvement in triglycerides and high‐density lipoprotein (HDL‐C). The reduction in low‐density lipoproteins (LDL‐C) was significant but similar to semaglutide.^154^ In combination with basal insulin, tirzepatide showed a strong reduction in HbA1c and weight. GI side effects were comparable to those of GLP‐1 RAs but were numerically greater at 15 mg compared with semaglutide 1 mg.^154^

**TABLE 3:** SURPASS program phase 3 trials with efficacy results

Trial	Design	*N*	Primary endpoint (weeks)	Background therapy	Comparator	Mean HbA1c at baseline (%)	Duration of diabetes (years)	Mean BMI at baseline (kg/m^2^)	Mean HbA1c reduction from baseline (%) with tirzepatide 5 mg/10 mg/15 mg	Mean reduction in body weight from baseline (kg) with tirzepatide 5 mg/10 mg/15 mg
SURPASS‐1^153^	Blinded	478	40	None	Placebo	7.94	4.7	31.9	1.87/1.89/2.07	7.0/7.8/9.5
SURPASS‐2^154^	Open label	1879	40	Metformin	Semaglutide	8.28	8.6	34.2	2.01/2.24/2.30	7.6/9.3/11.2
SURPASS‐3^155^	Open label	1444	52	Metformin ± SGLT2i	Insulin degludec	8.17	8.4	33.5	1.93/2.20/2.37	7.5/10.7/12.9
SURPASS‐4^156^	Open label	2000	52	Metformin ± SGLT2i or sulfonylureas	Insulin glargine	8.52	10.5	32.6	2.24/2.43/2.58	7.1/9.5/11.7
SURPASS‐5^172^	Blinded	475	40	Insulin glargine ± metformin	Placebo	8.31	13.3	33.4	2.11/2.40/2.34	5.4/7.5/8.8

Abbreviations: BMI, body mass index; HbA1c, haemoglobin A1c; *N*, number of clinical trial participants; SGLT2i, sodium–glucose cotransporter 2 inhibitors.

#### 3.2.3 Dual GLP‐1 receptor/glucagon receptor (GCGR) agonists

As described previously, essential functions of GLP‐1 RAs consist in delaying gastric emptying, stimulating insulin secretion and mediating satiety in the central nervous system, all beneficial effects for patients with obesity and/or T2D. A counterpart to GLP‐1 and insulin is glucagon. The peptide hormone is secreted by the alpha cells of the pancreas in response to fasting or hypoglycaemia. It stimulates gluconeogenesis and glycogenolysis, thereby increasing blood glucose levels.^157^

Consequently, the unimolecular co‐agonism of GLP‐1 and glucagon receptor (GCGR) for managing T2D and obesity seems counterintuitive at first. The reason for exploring this strategy lies in the additional catabolic and thermogenic actions of glucagon. It has been demonstrated that the intravenous administration of glucagon decreases plasma lipids and stimulates lipolysis in white adipocytes.^157^, ^158^, ^159^, ^160^ Its infusion also stimulates energy expenditure, characterized by increased oxygen consumption.^157^, ^161^, ^162^ Glucagon thermogenetic effects are mediated via increasing brown adipose tissue temperature^163^ and possibly futile substrate cycling.^164^

**FIGURE 2:**
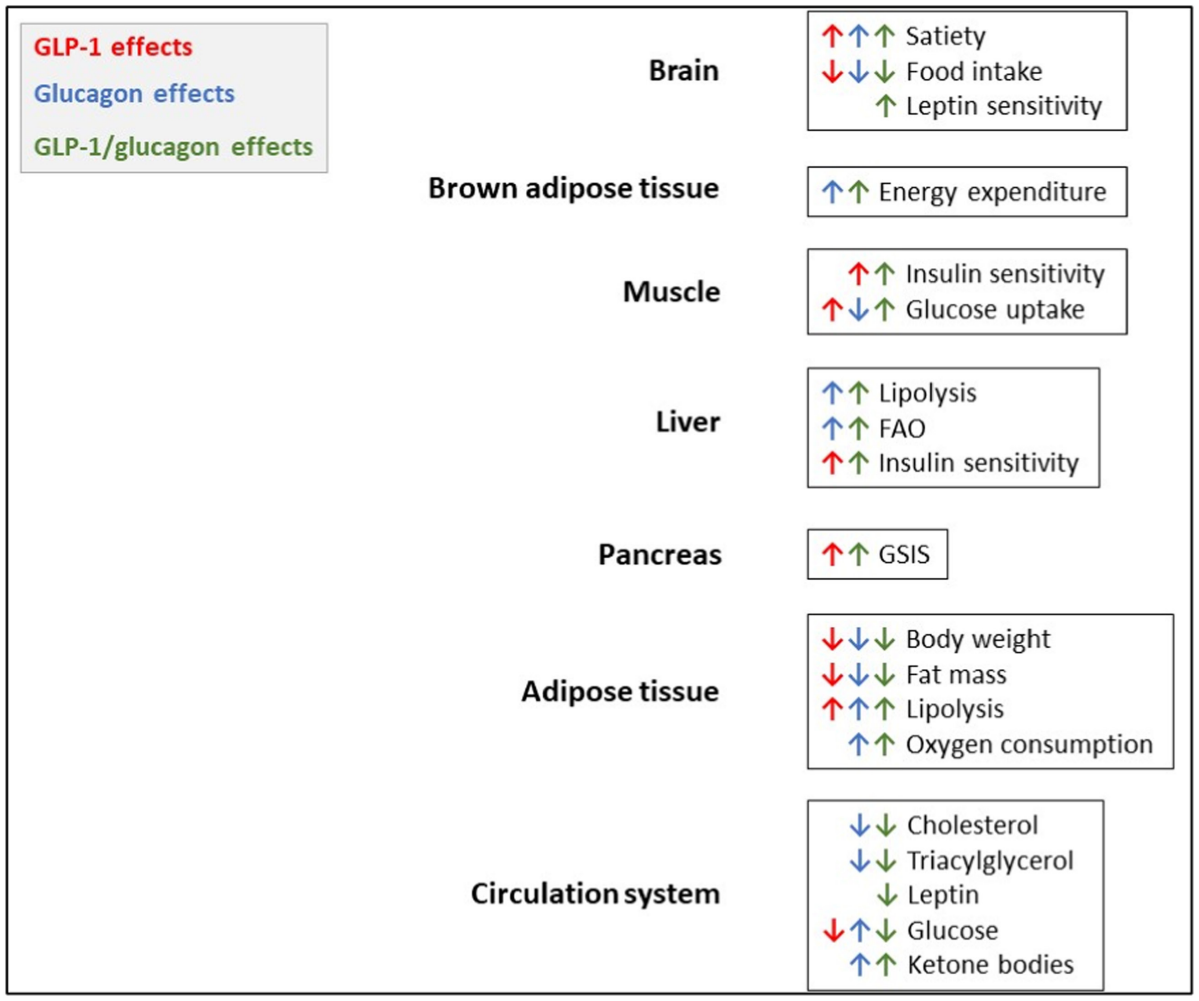
Physiological effects of GLP‐1 RAs, GCGR agonists and dual GLP‐1 R/GCGR agonists on different organs and tissues (pancreas, liver, brain, brown adipose tissue, muscle, adipose tissue and circulation system). FAO, fatty acid oxidation; GSIS, glucose‐stimulated insulin secretion). Adapted from 157

To address the thermogenic and catabolic mechanisms of glucagon that could be beneficial for persons with T2D and/or obesity, and at the same time avoid the gluconeogenesis and glycogenolysis stimulating effects of glucagon, GLP‐1 R/ GCGR co‐agonists are at present in development (Table 4). The effects of GLP‐1 RAs, GCGR agonists and GLP‐1 R/GCGR co‐agonists are depicted in Figure 2.

**TABLE 4:** List of dual GLP‐1/glucagon receptor co‐agonists currently in development and the respective indication

Drug	Company	Phase	Indication
Pemvidutide (ALT‐801)	Altimmune	Phase I	NASH/obesity/type 2 diabetes
HM12525A	Hanmi Pharmaceuticals (Collaboration with MSD)	Phase II	NASH
Cotadutide (MEDI0382)	AstraZeneca and MedImmune	Phase II	Type 2 diabetes/obesity/NASH/diabetic kidney disease
BI 456906	Boehringer Ingelheim (Collaboration with Zealand Pharma)	Phase II	NASH and liver fibrosis/obesity
IBI362	Innovent Biologics	Phase II	Obesity/diabetes
MK‐3655	Merck	Phase II	NASH
OPK88003	OPKO Health	Phase II	Type 2 diabetes/obesity

Note

In addition to type 2 diabetes and obesity, GLP‐1 R/GCGR co‐agonists are being developed for the indication non‐alcoholic steatohepatitis (NASH). Adapted from 157, 173, 174, 175.

#### 3.2.4 Triple GLP‐1/GIP/GCG receptor agonists

A further step along the way of combining peptide hormones into a single molecule would be to create a multifunctional incretin peptide with activity against three receptors.^165^ One such example was the GLP‐1/GIP/GCG RA with the goal of further reducing appetite and modulate energy expenditure to provide additional weight loss and improve health benefits.^166^ A first‐in‐human dose study in healthy volunteers with the triple agonist LY3437943^167^ demonstrated statistically significant dose‐dependent weight loss of up to 3.5 kg at the highest dose vs. placebo. Importantly, weight loss was maintained up to Day 43 following single administration of the two highest doses.^167^

#### 3.2.5 GLP‐1 RAs in combination with amylin mimetics

A human amylin analogue has been studied in phase 1b randomised controlled trial in obese people without diabetes with or without concomitant administration of the GLP‐1 RAs semaglutide 2.4 mg. The aim of the study was to determine the safety, tolerability, pharmacokinetics and pharmacodynamics of this drug combination. It has been shown that concomitant treatment with once‐weekly subcutaneous cagrilintide and once‐weekly subcutaneous semaglutide 2.4 mg was well tolerated with an acceptable safety profile. As expected with a GLP‐1 RA, the majority of treatment‐related adverse events were GI disorders. Moreover, the combination therapy proved effective for weight management. At week 20, three out of six doses of cagrilintide (1.2, 2.4 and 4.5 mg) in conjunction with 2.4‐mg semaglutide yielded significant weight loss (15.7%, 17.1% and 15.4%, respectively) compared to with semaglutide alone. In addition, glycaemic parameters improved in all treatment groups, regardless of the cagrilintide dose.^168^

## 4 CONCLUSION

Obesity and T2D are recognized as tightly interconnected concomitant diseases, associated with serious morbidity. As discussed in this paper, intentional weight loss can reverse T2D metabolic abnormalities and thus improve glycaemic control with additional benefits of improved cardiometabolic disease risk factors. While clinical benefits are typically set in upon achieving a weight loss of 5%, larger weight losses may lead to further health improvements. Furthermore, sustained weight loss of >15% can have a disease‐modifying effect in people with T2D, an outcome that up to recently could not be achieved with any blood glucose‐lowering pharmacotherapy. However, despite considerable therapeutic progress, there is still a large unmet medical need in patients with T2D who miss their individualized glycaemic and weight loss targets.

Thus, T2D treatment needs to consider multiple goals simultaneously in addition to glucose control, specifically weight management, cardiovascular and renal risk reduction and improved adherence. However, if we accept the validity of the emerging evidence, that obesity is upstream of T2D in vast majority of patients,^28^ then we believe that an early weight‐centric approach to T2D treatment would inevitably result in an effective and holistic approach to T2D with benefits in terms of the multiple T2D treatment goals simultaneously. In this sense, the available new therapeutic interventions can make a difference for our patients when broadly deployed. Advanced treatment options as the widely available GLP‐1 RAs have been shown to support holistic goals by lowering HbA1c without weight gain and hypoglycaemia, and importantly with great potential for reducing hard endpoints such as cardiovascular and kidney outcomes. The results of the combination therapies of GLP‐1RAs with SGLT2i, as well as those of the GLP‐1/GIP and GLP‐1/GCGR co‐agonists look increasingly promising, and it is to be expected that further development and use of these therapies will favourably change the scenario of weight and glucose control in T2D.

## CONFLICTS OF INTEREST

Prof. Mathias Blüher has received honoraria as a consultant and speaker from Amgen, AstraZeneca, Bayer, Boehringer Ingelheim, Daiichi‐Sankyo, Lilly, Novo Nordisk, Novartis, Pfizer and Sanofi. Prof. Antonio Ceriello has been involved in Advisory Boards, Lectures and Consultancy by the following companies: Astra Zeneca, Berlin Chemie, Eli Lilly, Novo Nordisk, Italian Ministry of Health—Ricerca Finalizzata, Mitsubishi, Roche Diagnostics and Theras. Dr. Helena Rodbard has consulted for and received research support by Bayer, Boehringer Ingelheim, Eli Lilly, Merck, Novo Nordisk, Regeneron, Sanofi, Vivus and Zealand. Prof. Naveed Sattar has consulted for Afimmune, Amgen, AstraZeneca, Boehringer Ingelheim, Eli Lilly, Hanmi Pharmaceuticals, Merck Sharp & Dohme, Novartis, Novo Nordisk, Pfizer and Sanofi; and received grant support paid to his University from AstraZeneca, Boehringer Ingelheim, Novartis and Roche Diagnostics outside the submitted work. Prof. Oliver Schnell is a founder and CEO of Sciarc GmbH. Elena Tonchevska is an employee of Sciarc GmbH. Prof. Melanie Davies and Prof. Fracesco Giorgino have not reported any conflicts of interest.

## AUTHOR CONTRIBUTIONS

**Matthias Blüher:** Conceptualization (equal); Methodology (equal); Supervision (equal); Writing – original draft (equal); Writing – review & editing (equal). **Antonio Ceriello:** Conceptualization (equal); Methodology (equal); Supervision (equal); Writing – original draft (equal); Writing – review & editing (equal). **Melanie Davies:** Conceptualization (equal); Methodology (equal); Supervision (equal); Writing – original draft (equal); Writing – review & editing (equal). **Helena Rodbard:** Conceptualization (equal); Methodology (equal); Supervision (equal); Writing – original draft (equal); Writing – review & editing (equal). **Naveed Sattar:** Conceptualization (equal); Methodology (equal); Supervision (equal); Writing – original draft (equal); Writing – review & editing (equal). **Oliver Schnell:** Conceptualization (equal); Methodology (equal); Supervision (equal); Writing – original draft (equal); Writing – review & editing (equal). **Elena Tonchevska:** Conceptualization (equal); Methodology (equal); Project administration (lead); Writing – original draft (equal); Writing – review & editing (equal). **Francesco Giorgino:** Conceptualization (equal); Methodology (equal); Supervision (equal); Writing – original draft (equal); Writing – review & editing (equal).

## Data Availability

Data sharing is not applicable to this article as no new data were created or analysed in this study.
